# Hispanic Thrifty Food Plan (H-TFP): Healthy, Affordable, and Culturally Relevant

**DOI:** 10.3390/nu16172915

**Published:** 2024-09-01

**Authors:** Romane Poinsot, Matthieu Maillot, Adam Drewnowski

**Affiliations:** 1 MS-Nutrition, 13005 Marseille, France; romane.poinsot@ms-nutrition.com (R.P.); matthieu.maillot@ms-nutrition.com (M.M.); 2 Center for Public Health Nutrition, University of Washington, Seattle, WA 98195, USA

**Keywords:** thrifty food plan, National Health and Nutrition Examination Survey (NHANES), Hispanic population, Healthy Eating Index 2015, quadratic programming, pork, affordable diet

## Abstract

The USDA Thrifty Food Plan (TFP) is a federal estimate of a healthy diet at lowest cost for US population groups defined by gender and age. The present goal was to develop a version of the TFP that was more tailored to the observed dietary patterns of self-identified Hispanic participants in NHANES 2013–16. Analyses used the same national food prices and nutrient composition data as the TFP 2021. Diet quality was measured using the Healthy Eating Index 2015. The new Hispanic TFP (H-TFP) was cost-neutral with respect to TFP 2021 and fixed at $186/week for a family of four. Two H-TFP models were created using a quadratic programming (QP) algorithm. Fresh pork was modeled separately from other red meats. Hispanic NHANES participants were younger, had lower education and incomes, but had similar or higher HEI 2015 scores than non-Hispanics. Their diet included more pulses, beans, fruit, 100% juice, grain-based dishes, and soups, but less pizza, coffee, candy, and desserts. The H-TFP market basket featured more pork, whole grains, 100% fruit juice, and cheese. The second TFP model showed that pork could replace both poultry and red meat, while satisfying all nutrient needs. A vegetarian H-TFP proved infeasible for most age–gender groups. Healthy, affordable, and culturally relevant food plans can be developed for US population subgroups.

## 1. Introduction

The Thrifty Food Plan (TFP), developed by the U.S. Department of Agriculture (USDA), is a mathematical tool used to calculate the lowest cost of a healthy diet at home [1,2]. The TFP market basket follows the Dietary Guidelines for Americans (DGA), respects existing eating patterns, and meets pre-set requirements for nutrient adequacy and for lowest cost [1,2]. The estimated cost of the TFP market basket is highly important since it is used to calculate benefit amounts under the Supplemental Nutrition Assistance Program (SNAP) [3]. The last revision of the TFP was conducted in 2021 by the USDA Center for Nutrition Policy and Promotion (CNPP) [2].

Population subgroups that self-identify as Hispanic may have distinct eating patterns that provide adequate nutrition at an affordable cost [4]. Currently, there is no TFP that is specifically tailored to the needs of Hispanic or any other population subgroup; rather, the Dietary Guidelines emphasize that all individuals and households can make food choices that are both budget-sensitive and culturally appropriate [5]. For the Hispanic population, the default is toward using staple foods to create affordable and nutritious meals [6]. Staple foods thought to be common in Hispanic cuisine include fresh vegetables, seafood, tortillas, rice, corn, and beans [6].

However, cultural stereotypes are not necessarily true. Whether those foods are regularly consumed by Hispanic participants in the National Health and Nutrition Examination Survey (NHANES) is a good question [7,8,9]. Our first goal was to characterize the observed eating habits of the Hispanic population in the two cycles of the National Health and Nutrition Examination Survey (NHANES 2013–16) [10]. To undertake that, we needed to identify those NHANES 2013–16 participants who self-identified as Hispanic, describe their existing eating habits, and develop population-specific estimates of household diet quality in relation to diet cost. Those would be the input data for a Hispanic Thrifty Food Plan (H-TFP), a cost-neutral market basket that would respect existing eating patterns of the Hispanic NHANES sample.

Our analytical methods closely followed those used by the USDA/CNPP to develop the revised TFP 2021 [2]. Those protocols are documented in supplemented datafiles published online [2]. The modeling categories came from the USDA TFP 2021, except that fresh pork was treated as a separate meat category [11]. Nutrient composition data came from the USDA Food and Nutrient Database for Dietary Studies (FNDDS 2015–16) [12]. National food prices, adjusted to June 2021, were the same as those used in the TFP 2021 [13]. The US-Style Healthy Diet Pattern [14] was the selected diet quality standard, same as previously [11]. The cost constraint for the H-TFP was set at $189.91, which was the cost estimated in a previous study [11] and very close to the USDA-revised TFP 2021 [2].

The main goal was to develop a cost-neutral H-TFP that would comply with the USDA Healthy US-Style Food Pattern, while meeting nutrient requirements and following the specific eating patterns of the Hispanic population in the NHANES sample. A second goal was to test the feasibility of modeling a vegetarian H-TFP.

## 2. Materials and Methods

### 2.1. Analyses of Dietary Intakes

#### 2.1.1. National Health and Nutrition Examination Survey (NHANES) 2015–2016

The nationally representative National Health and Nutrition Examination Survey (NHANES) is an ongoing survey of dietary intakes and health outcomes in the US. The present analyses were based on two cycles of NHANES (NHANES 2013–16) for a total of 13,826 participants aged >4 y, 4180 of whom self-identified as Hispanic. The categorization was based on the race–ethnicity variable in the NHANES demographic data. In the NHANES 2013–16, the Hispanic population can be disaggregated into Mexican Americans and other Hispanics.

What We Eat in America (WWEIA) studies are the dietary intake component of the NHANES. The present estimates of the US population’s eating habits came from the 1st-day dietary recalls in the NHANES 2013–16. Mean consumption patterns were calculated for all age–gender groups. Gender was defined as male and female. Age groups were 4–13 y, 14–19 y, 20–50 y, and 51–70 y. For calculating TFP costs, our reference family of four was two adults aged 31–50 y and two children aged 4–13 y. For comparison purposes, the USDA TFP 2021 [2] stratified the population into 15 groups by age and gender, from age 1 y to age 74 y. The family of four in TFP 2021 was two adults aged 31–50 y, one child 6–8 y, and one child 9–11 y.

#### 2.1.2. Healthy Eating Index HEI-2015

The HEI-2015 score, a measure of compliance with Dietary Guidelines for Americans [15], was calculated for each NHANES participant. The HEI 2015 score is a 100-point scale with 13 components that contribute from 5 to 10 points to the total score. The components that are scored positively are as follows: Total Vegetables, Greens and Beans, Total Fruit, Whole Fruit, Whole Grains, Dairy, Total Protein Foods, Seafood and Plant Protein, and Fatty Acid Ratio. Points are given for low consumption of Refined Grains, Saturated Fats, Added Sugars, and Sodium. Higher HEI-2015 scores indicate better alignment with the Dietary Guidelines.

An unweighted median HEI-2015 score was then estimated for males and females in each age group. Following USDA protocols for TFP 2021, the “observed” diets were only those with HEI-2015 scores above the group median. Only those diets were the input variables in the H-TFP modeling analyses. This was to ensure that nutrient-dense food items were included in the optimization model. The more nutrient-dense foods tend to be consumed by individuals with higher HEI-2015 scores.

#### 2.1.3. Plan of Analysis for Dietary Intakes Data

Firstly, the Hispanic and non-Hispanic populations in the NHANES 2013–16 were characterized by socio-demographic variables: gender, age group, education, incomes, and FIPR (family income to poverty ratio). Secondly, mean consumption patterns, expressed as the weekly intakes of combined categories for a family of four, were compared between Hispanic and non-Hispanic groups. The mean consumption for the 4-person family was the sum of the mean consumptions of adult man/woman and children female/male. Thirdly, estimates of the Healthy Eating Index (HEI-2015) were compared by group, gender, and age group.

### 2.2. Input Data for Quadratic Programming (QP) Optimization Model

In QP optimization models, the QP algorithm tries to minimize the objective function, which was the deviation from the observed diets. This dietary deviation was defined as the sum of the square root of deviation between the observed and optimized amount for each combined category. Each combined category deviation was standardized by the observed amount and was weighted by the cost contribution of the combined category to the total observed diet cost. In other words, the objective function was calculated as the mathematical distance between the observed and optimized diets weighted by the cost contribution of combined categories and expressed in % deviation from the observed. Contrary to expectations, the USDA TFP optimization model did not start with the observed diets of the entire population; rather, the observed diets, the principal input into the QP model, were carefully selected as those with HEI values above the median value for each population subgroup. Selecting diets with above-median HEI scores was reportedly performed to guarantee the incorporation of more nutrient-rich food items into the QP optimization model. However, those “healthier” diets were no longer representative of the entire population. Diets with above-median HEI scores tend to have more leafy green vegetables and whole fruit, more plant-based proteins and seafood, and substantially less meat. It is important to know that contrary to most optimization models reported in the literature, the observed number of combined categories, used as a starting point of the USDA TFP optimization, was not based on average daily intakes but rather on average portion consumed at a meal by participants with the healthiest diets.

The present analyses followed the USDA TFP 2021 approach, except that fresh pork was a separate modeling category. Following the USDA protocol exactly, the “observed” diets, used as input for the optimization model, were those diets that were above the median HEI-2015, with HEI values established for each Hispanic population subgroup.

#### 2.2.1. Food and Nutrient Database for Dietary Studies (FNDDS 2015–16)

Individual foods and beverage items in the FNDDS 2015–16 are identified by WWEIA food codes [16]. The USDA TFP 2021 had already assigned FNDDS food items to 1 of 65 initial modeling categories. Those TFP categories were largely based on What We Eat in America (WWEIA) 4-digit codes [16] with some modifications by the USDA/CNPP [2]. Selected grains, fruit, dairy, protein foods (including meats), popcorn, and beverages were split into subcategories of higher or lower nutrient density as determined by their content of sodium, saturated fat, and sugar (see Supplemental Table S1).

Higher nutrient density meats with saturated fat content of <4.5 g/100 g were separated into beef and pork (higher nutrient density) [2,11]. Similarly, lower nutrient density meats were separated into beef and pork (lower nutrient density). This increased the number of TFP initial categories from the original 65 to 67. Aggregating initial modeling categories yielded a total of 46 so-called combined categories.

#### 2.2.2. National Food Prices from the USDA

National food prices for 3072 foods and beverages came from the USDA supplemental datafiles [2]. The pricing strategies were described in the TFP 2021 report.

The 2015–2016 Purchase to Plate Price Tool provides the mean national retail prices for 3231 FNDDS food codes, i.e., for almost 97% of food and beverages reported in the What We Eat in America (WWEIA) studies [2]. The prices came directly from retailers and were adjusted for inflation to June 2021 by the USDA/CNPP. In developing the TFP 2021 [2], the USDA excluded from analysis infant formula and food codes with small sample sizes (159 food codes). Price outliers, such as expensive lobster or lamb, were also excluded from the calculations of modeling category prices. The TFP 2021 created higher and lower price categories, based on the 35th percentile cut point, for 30 out of 65 modeling categories. Food and beverage choices of higher-income households (>350% of federal poverty) were also excluded from the modeling of weighted category prices.

Following the USDA/CNPP methodology, inflation-adjusted food prices were merged with the FNDDS nutrient composition data and mean-weighted prices were then calculated for each TFP food category and combined category, as described above. Missing prices and price outliers were excluded from calculations of the weighted mean or, in other words, had a weight of zero. Outliers were defined as items with a price above the 1.5 interquartile ranges above the 1st quartile of each TFP 2021 category and subcategory.

#### 2.2.3. Population-Specific Weighted Modeling Variables

The mean nutrient composition of each QP modeling category was weighted by observed patterns of consumption. For example, among higher nutrient density meats in the TFP were many cuts of fresh pork: pork chop (baked, broiled, stewed, fried), pork roast, and pork steak/cutlet. Lower nutrient density meats (with saturated fat > 4.5 g/100 g) were pork roast, pork spareribs with barbecue sauce, ground pork, pork steak/cutlet (baked, broiled), and pork chop (breaded and baked, broiled, fried). Consumption patterns for these items can vary between Hispanic and non-Hispanic groups. As an example, the Hispanic subgroup did not consume any pork chops. The weighted nutrient content for each modeling category was determined by population-specific consumption patterns. Similarly, the weighted prices for each modeling category were determined by consumption patterns. As an example, the Hispanic subgroup might consume different types of fruit, which would be reflected in different prices paid for that modeling category.

Following TFP 2021, we adjusted for plate waste and/or leftovers. A food waste adjustment factor of +5% was applied to each modeling category for the 8 age–gender groups.

#### 2.2.4. Cost and Nutrient Density of Modeling Categories

In the TFP 2021, foods in each market basket group were divided into high-cost and low-cost food items. Animal protein foods (beef, pork, poultry, and seafood) were split into low-cost and high-cost food items and so were dairy products. Cured meats, eggs, nuts, and soy were not split by food cost.

In the present analyses, the nutrient density of the market basket groups was established using a nutrient density score that was based on the 24 nutrients for which TFP 2021 constraints had to be met: carbohydrate, fat, protein, fiber, calcium, copper, linoleic acid, alpha-linolenic acid, iron, folic acid, potassium, magnesium, zinc, niacin, phosphorus, riboflavin, thiamin, vitamin E, vitamin A, vitamin B12, vitamin B6, vitamin C, vitamin K, and choline. The NDS24 score, expressed as %/100 kcal, was the mean percentage of adherence to Recommended Nutrient Values (RNVs) per 100 kcal. The standards used were for males, aged 20–50 y. If NSD24 = 5%/100 kcal, it means that the food can provide on average 100% of the recommended values for 2000 kcal.

We then compared NDS24 values, energy density (kcal/100 g), and prices for market basket groups (including the disaggregated protein group) and high-cost/low-cost/other groups between Hispanic and non-Hispanic populations. Firstly, foods assigned by the USDA into the lower-cost categories were indeed lower cost. However, for the Hispanic group, lower cost was not necessarily linked to lower nutritional value. In the protein foods category, lower-cost pork items were associated with higher nutrient density scores. This has implications for optimization analyses that try to maximize nutritional value at affordable cost. The data are summarized in Supplemental Table S2.

#### 2.2.5. Healthy US-Style Food Pattern

The Healthy US-Style Dietary Pattern [14] was the source of minimal and maximal amounts of diverse food groups and subgroups within each calorie level. Modeling categories were translated into quantities of food groups and subgroups of interest with the Food Patterns Equivalent Database (FPED) 2015–16 [17]. Following USDA methodology, minimum amounts were specified for vegetables, fruits, grains, dairies, total protein foods, and oils, and for dark green vegetables, red and orange vegetables, beans/peas/lentils, starchy vegetables other vegetables and whole grains, meats/poultry/eggs, seafood, and nuts/seeds/soy products. One half or more of the fruit group had to come from whole fruit and one half or more of the total grains had to come from whole grains. One half or more of total dairy had to come from the higher nutrient density milk and yogurt.

Maximum amounts for the food groups and food subgroups were set at the 95th percentile of reported dietary intake. Refined grains were an exception; here, a fixed amount was defined, e.g., 5 oz eq/d for male 20–50 y. In those cases where the 95th percentile was below the recommended amount, the maximum was set at the recommended amount plus 10%. For those food groups that did not directly correspond to a Healthy US-Style Dietary Pattern component, the lower bound was the 25th percentile of reported dietary intake and the upper bound was the 95th percentile (or 75th percentile for eggs) of reported dietary intake.

### 2.3. Quadratic Programming (QP) Optimization Models

**Figure 1:**
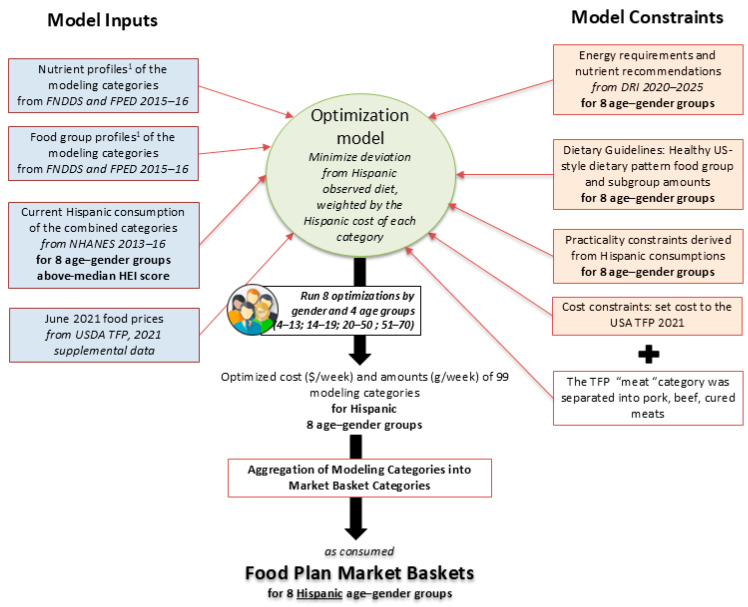
A schema of the quadratic programming optimization model, showing model inputs and model constraints. ^1^ weighed by Hispanic consumptions.

The QP optimization model closely followed the methods used by the USDA/CNPP to develop TFP 2021 (Figure 1).

Firstly, national food prices were the same as had been used in the TFP 2021 revision. The cost constraint was set at $189/week for a family of 4, close to the TFP 2021 values. Observed consumption patterns for the Hispanic participants came from the NHANES 2013–16 dietary intake database. Following USDS procedures, we used only those diets with above-median HEI scores for each population subgroup. The minimum and maximum bounds for each food group came from the USDA Healthy US-Style Dietary Pattern [14]. Nutrient standards came from the National Academies (NASEM) [18]. A QP optimization model is defined by a set of input variables, a list of constraints, and an objective function that needs to be optimized (i.e., minimized or maximized) [11,19,20]. The input variables were the quantities *x_i_* of each modeling category *i*, *i* = 1, …, 67. The objective function of the QP model was a quadratic function that minimizes the overall distance between the quantity *x_j_* of the combined category *j*, and the average consumption *c_j_*, weighted by the expenditure shares of each combined category. Constraints ensure the diet meets established nutritional standards and adheres to recommended minimum and maximum bounds for each food group. Practicality constraints address specific categories, such as ensuring that at least half of the grains are whole grains and that a significant portion of dairy comes from higher nutrient density options like milk and yogurt. For each model, the cost constraints were the same as in the TFP 2021 ($189/week for a family of 4).

- Model 1, H TFP: This model determined the composition of a “cost neutral” H-TFP market basket.
- Model 2, H-TFP pork: In this model, pork replaced beef and poultry and was the only source of meat. The goal was to determine whether a TFP with pork as the only source of meat would conform to Dietary Guidelines and satisfy all nutrient needs with no increase in cost. The permitted amounts of the modeling categories of beef, poultry, and cured meat were each set to zero. The constraints on having minimal and maximal amounts of meat and poultry (as per USDA Healthy US-Style Food Plan) were removed. The amounts of seafood, eggs, and nuts and seeds were determined by the QP algorithm. The QR algorithm then searched for a healthy food plan that would satisfy all nutrient requirements. In this model, cost was allowed to increase by 0.07 to achieve a feasible mathematical solution for females aged 51–70 y.
- Model 3, Vegetarian: In this model, the permitted amounts of the modeling categories of beef, poultry, cured meat, seafood, and mixed dishes with meat, poultry, or seafood were all set to zero. The requirement to include a minimum amount of seafood (per US-Style Healthy Food Plan) was removed. The requirements to include minimum amounts of “meat + poultry + egg” and total protein foods were set to 0. The range for eggs was set at between 6 and 25 g/day. The QP algorithm then searched for cost-neutral vegetarian market baskets that would satisfy all energy and nutrient requirements.

Modeling categories were the same as in the TFP 2021. Following past procedures, pork was treated as a distinct modeling category, separate from other non-poultry meats. For each modeling category, mean nutrient composition and mean price were weighted by the eating habits of this particular population subgroup. Separate modeling analyses were conducted for 8 age–gender groups. The amounts and estimated weekly costs were calculated for a reference family of 4. The three QP models are summarized in Table 1.

The overall optimization scheme is shown in Figure 1. Our procedures were very similar to those used to develop the revised USDA TFP 2021.

### 2.4. Statistical Analysis

Food amounts were converted from daily to weekly quantities by multiplying them by seven. Weekly costs were estimated for the entire diet and food categories “as consumed”. Observed and optimized amounts and costs, determined by category, were estimated for a family of four, consisting of the sum of one male aged 20–50 years, one female aged 20–50 years, one male aged 4–13 years, and one female aged 4–13 years. For comparison, the USDA reference family of four includes an adult male, an adult female, and two children aged 6–8 years and 9–11 years.

To ensure sample representativeness, comparisons on observed consumptions accounted for the NHANES sampling frame design. Chi-square tests were conducted to examine potentially significant associations between socio-demographic characteristics and population origin. HEI per age–gender group was compared between Hispanic and non-Hispanic using *t*-test. Observed consumption and costs of the family of four were compared between Hispanic and non-Hispanic. As observed consumption for the family is the sum of the mean consumption of four age–gender groups, *t*-tests were performed on each group, and the results indicated the number of groups for which the *p*-value was significant. Two different methods for estimating observed consumption were presented as follows: the first is based on the entire population, not selected by HEI scores, and the second uses the mean serving sizes for foods consumed by NHANES study participants with above-median HEI scores (the method used to define current consumption in the optimization model). Statistical analyses used the R software version 4.4. The level of significance was set to 5% for all tests.

## 3. Results

### 3.1. Socio-Demographic Characteristics

Characteristics of the Hispanic and non-Hispanic populations in the NHANES 2013–16 sample are summarized in Table 2. The Hispanic population (*n* = 4180) was composed of Mexican Americans (53%) and other Hispanics (37%). The Hispanic population was generally younger, with lower education and incomes and with significantly lower family income-to-poverty ratios. The degree of acculturation was not examined [9,21].

### 3.2. Diet Quality Measured by HEI-2015 Scores

Median and mean HEI-2015 values for the Hispanic and non-Hispanic groups by age and gender are shown in Table 3. Despite major differences in socioeconomic status, the HEI 2015 scores were not significantly different. For one group (females aged 4–13 y), the HEI-2015 score was higher (49.6 vs. 47.6) compared to the non-Hispanic group.

### 3.3. Observed Consumption Patterns (g/week) and Estimated Diet Costs ($/week) for Hispanic and Non-Hispanic Populations in NHANES 2013–16: Data for a Family of 4

Table 4 shows the amounts (in g/week) and estimated food costs (in $/week) for Hispanic and non-Hispanic HNANES 2013–16 participants. The data are for a family of four, as previously defined. The food groups were the combined TFP 2021 categories, defined by the USDA, and close to the USDA market basket categories.

Firstly, total consumption of vegetables was comparable for the two population groups. The Hispanic group consumed less dark green and red/orange vegetables but much more beans, peas, and lentils (466 g vs. 94 g). The consumption of fruit juice was substantially higher in the Hispanic group (an additional 700 g per week). For grains, the consumption of staple grains (whole and refined breads, tortilla, bagels, pasta, noodles, rice) was higher, but the consumption of biscuits, muffins, and quick breads was lower. The consumption of milk and yogurt was similar but cheese was lower. For protein foods, the consumption of cured meats and nuts and seeds was lower, whereas the consumption of mixed egg dishes (omelets, scrambled eggs) was higher. In the miscellaneous category, the Hispanic group consumed more mixed dishes with beans and grains, less mixed dishes with meat or seafood, less mixed dishes with vegetables, and less pizza. The consumption of soups and sandwiches was higher; the consumption of candy, snack bars, coffee, and tea was lower.

The estimated diet cost for the observed diet for a family of four in the Hispanic group was $190.12, compared to $196.16 in the non-Hispanic group. The estimated costs were lower for vegetables, higher for fruits (both whole fruit and 100% fruit juice), and lower for dairy, grains, and protein foods. The overall costs were higher for miscellaneous foods, with higher costs for grain mixed dishes and lower costs for pizza. As noted above, the mean and median HEI-2015 scores for the two groups were comparable, denoting equivalent compliance with the Dietary Guidelines for Americans.

### 3.4. Observed Serving Sizes and Their Estimated Cost for Diets with Above-Median HEI Scores for Hispanic and Non-Hispanic Populations in NHANES 2013–16: Data for a Family of 4

The “observed” input diets used to develop the TFP 2021 were those with HEI 2015 scores above the median for each population subgroup. The amounts shown in Table 5 are not amounts consumed per week; rather, those are mean portion or serving sizes for those foods that were consumed by those NHANES study participants with above-median HEI scores. Those data were the input for the TFP 2021; the same calculations are now recreated for the Hispanic and non-Hispanic population subgroups.

There are some differences between the observed diets and the observed “healthier” diets that are consistent with higher HEI scores. The healthier diets with above-median HEI scores have larger amounts of vegetables, including dark green vegetables, lower amounts of staple grains, lower amounts of cured meat and nuts and seeds, and much lower amounts of fruit-based drinks and sodas. The estimated cost was around $120 per week for a family of four. Those data served as the input for the H-TFP, following the USDA approach exactly.

### 3.5. H-TFP Optimization Models Compared to the TFP 2021

The H-TFP (Model 1) and H-TFP pork (Model 2) were then compared to the previously published TFP for the US population [11]. Shown in Table 6 are the amounts per week (g/week) and prices per week ($/week) for a family of four. Both H-TFP models were designed to conform to the US-Style Healthy Food Pattern by gender and age group. We observed, for example, that the present H-TFP and H-TFP pork models were closer to the observed intakes for the Hispanic group than was the USA-TFP (Supplemental Table S3).

The optimized food patterns in the H-TFP had the same amounts of total vegetables as in the TFP 2021. The amounts of dark green and red/orange vegetables and beans, peas, and lentils were very similar. The H-TFP had more starchy vegetables and less other vegetables. The optimized amount of fruit was the same for TFP 2021 and for H-TFP, but the H-TFP model selected a higher proportion of 100% fruit juice, more consistent with observed eating habits. The H-TFP had substantially higher amounts of whole-grain cereals, including oatmeal and RTE cereals, and less refined grains. The dairy group in the H-TFP had no low nutrient density (i.e., sweetened) dairy but comparable amounts of high nutrient density dairy and much more cheese.

Our version of the TFP 2021 model [11] selected pork in preference to other red meats and did not select any cured meat. The H-TFP selected even higher amounts of pork and substantially less poultry. Both seafood and nuts and seeds were at their maximum allowed limit.

The amount of the miscellaneous was higher among H-TFP than TFP 2021, and the difference came from the “Other Miscellaneous Foods and Beverages” market basket (218 d/week for TFP 2021 vs. 2439 g/week for H-TFP). H-TFP had more nutritional beverages (990 g/week for Hispanic vs. 0 g/week) but also more soda, both regular (1106 g/week for Hispanic vs. 0 g/week) and diet (300 g/week for Hispanic vs. 0 g/week).

Model 2 in Table 6 shows the result of the QP optimization where fresh pork was the only meat, replacing poultry, cured meat, and other red meat. Firstly, a cost-neutral mathematical solution was obtained, showing the fresh pork could be a major protein component of a healthy diet. All nutrient requirements were met. Amounts of seafoods, nuts, and eggs were constrained by the need to adhere to the USDA Healthy US-Style Food Pattern. Model 2 raised the amount of fresh pork to 3259 g/week (for a family of four), increased the amount of dairy, and reduced the amounts of grains including whole grains. There was a sharp reduction in the miscellaneous food group (especially the “Other Miscellaneous Foods and Beverages”).

### 3.6. Distribution of H-TFP Market Basket Costs

**Figure 2:**
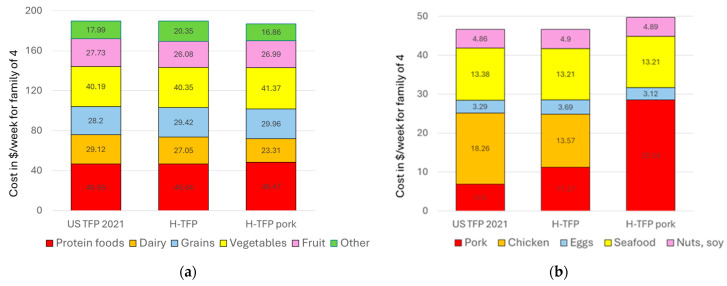
Food plan costs for all foods groups (**a**) and for the protein food group (**b**) in $/week by model and market basket group for a family of four.

The H-TFP was purposely set to be cost-neutral. The goal was to determine whether a version of the TFP could be constructed for a specific subpopulation at the same price. Figure 2 shows that the cost distribution was similar across the three different models. The costs for eggs, seafood, nuts, seeds, and soy products were the same because their consumption was prescribed by the pre-set minima and maxima in the USDA Healthy US-Style Food Pattern.

### 3.7. A Vegetarian H-TFP Was Not Mathematically Feasible

Many foods thought to be common in Hispanic cuisine are plant-based and include fresh vegetables, rice, corn, and beans [6], which is one message of the Old ways Latin Heritage Diet [6]. Accordingly, an attempt was made to create a vegetarian H-TFP market basket that was both nutrient-adequate and cost-neutral, i.e., equal in cost to the revised USDA TFP 2021.

Modeling analyses by gender and age group showed that there was no feasible mathematical solution for adolescent and adult males (ages 14–70 y). This was due in large part to minimum energy requirements that could not be met, given food group constraints. At the same time, several food groups were at the maximum levels recommended by the US-Style Healthy Food Patterns. Those were dairy products, eggs, legumes, nuts and seeds, dark green vegetables, and other vegetables. Several combined categories were also at maximum values, especially for men 20–50 y old. Those included alternative energy sources, as follows: butter and oils, crackers, desserts, popcorn, potatoes, and mixed dishes with grains, vegetables, and beans. The minimum requirement for alpha-linoleic acid and choline could not be met either. Any potential solution for adult males aged 20–50 y and 51–70 y required relaxing food group constraints and an increase in H-TFP cost of up to $10 per person per week.

For male and female adolescents (ages 14–19), no mathematical solution was obtained even after increasing the H-TFP market basket cost. The minimum energy requirement for boys could not be met. Several food categories were at maximum levels, notably eggs and dairy, legumes, and vegetables. Several combined categories were also at maximum levels, notably sweet bakery products, sandwiches, snack bars, and desserts. Maxima were achieved for pizza (girls) and sugar (boys). The vegetarian food plan also failed to deliver sufficient alpha-linoleic acid and choline.

A mathematical solution was obtained for females aged 20–70 y and 51–70 y and for children aged 4–13 y but at the expense of some very large deviations from the existing diets. The deviations from the existing diets are shown in Supplemental Table S3. Women and children had lower energy requirements that could be satisfied by a meatless vegetarian diet. However, the mathematical solution was achieved by replacing nutrients in meat not only with eggs and dairy but also pizza, sandwiches, sweet bakery products, and mixed dishes with eggs, vegetables, and beans.

## 4. Discussion

The USDA TFP is one of four official USDA food plans designed to model healthy diets at successively higher cost levels. The least costly TFP calculates the lowest cost of a healthy diet using current food prices and quadratic programming diet optimization methods. The estimated lowest cost of the TFP market basket is then used to calculate benefits under the Supplementary Nutrition Assistance Program (SNAP). While the TFP is not tailored to the needs of specific population subgroups, it is meant to be culturally sensitive and to accommodate different dietary habits and preferences [1,2].

The present goal was to develop a Hispanic version of the TFP that better followed the eating patterns of the Hispanic subgroup in the 2013–16 National Health and Nutrition Examination Survey (NHANES). The Hispanic population reached 62.1 million in 2020 in the US, accounting for 19% of the total and making it the nation’s second-largest racial or ethnic group [22]. Though recent studies on diet quality of that group are relatively few [7,8,9], they point to specific dietary habits and preferences. To our knowledge, culturally relevant versions of the TFP 2021 have not previously been created for specific subpopulations in the US.

This examination of the eating habits of NHANES 2013–16 participants was based on the first 24 h recall. Firstly, consistent with other studies [23], we showed that the group self-identified as Hispanic had lower incomes, lower poverty-to-income ratios, and lower education compared to the rest of the NHANES sample. On the other hand, HEI 2015 scores for the Hispanic group were comparable to the non-Hispanic population and were in one case significantly higher. The overall median HEI 2015 scores were around 50, meaning that diet quality still needed improvement, based on the usual USDA cut points. Interestingly, the Hispanic group achieved comparable diet quality at a somewhat lower estimated cost, consistent with some previous observations [4,24]. The finding that comparable diet quality could be achieved at lower cost led to a further examination of specific eating habits of the Hispanic subgroup.

Based on the present analyses, the eating habits of the Hispanic group failed to conform to some stereotypical assumptions. The data were presented for a family of four, to better align with the TFP 2021 report. There was no evidence for higher consumption of vegetables, whether dark green or red/orange, rice, or corn. There was no evidence for higher consumption of seafood, tortillas, chicken, eggs, or cheese. The consumption of nuts and nut butter was lower compared to the non-Hispanic group. On the other hand, we did observe higher consumption of beans, grain mixed dishes, and soups. The Hispanic group also consumed more fruit, generally in the form of 100% fruit juices rather than whole fruit. We also observed lower consumption of pizza, candy, and desserts but no differences in the consumption of sugary soda or of fruit-based drinks.

As noted above, the USDA TFP 2021 optimization models were not based on the population-wide diets; rather, the “observed” diets taken as the input for TFP modeling were only those with HEI 2015 scores above the median value for each population subgroup. Selecting “healthier” diets as the departure point for optimization modeling necessarily maximizes the amounts of vegetables, whole fruit, and whole grains and minimizes the amounts of added sugars, solid fats, refined grains, and meat. The present modeling analyses for the H-TFP directly followed the USDA CNPP approach.

We developed two versions of the H-TFP. Our procedures closely followed those used by the USDA to develop the TFP 2021. We used the same nutrient composition and dietary intakes databases, the same national food prices, and a comparable quadratic programming (QP) optimization model. The main features of our H-TFP modeling were as follows. Firstly, the cost was deliberately set to be the same as the TFP 2021. The new H-TFP versions were cost-neutral, meaning that the cost for a family of four was set at $189, the value obtained in a previous study [11] and very close to the TFP 2021. Secondly, pork was separated from the other red meats, same as previously. Consistent with the TFP 2021, data on market basket amounts and cost were presented for a family of four.

The new H-TFP market basket featured more whole grains, more fresh pork, and less poultry, compared to the previously published TFP for the US population [11]. Since the Hispanic population often selected different foods from within a given food category, the prices paid for some food categories were not the same.

In the second version of the H-TFP, fresh pork replaced other red meat and poultry and was the only source of meat. All the nutrition and practicality requirements were met and the H-TFP market basket remained cost-neutral. The goal was to show that healthy diets on a budget could be constructed with fresh pork as the only source of meat. The second model showed that the diet met energy and nutrient constraints and was mathematically feasible—but there were deviations from existing diets especially for women. The amounts of eggs, seafood, and nuts had been pre-set by the USDA Healthy US-Style Food Patterns and were not affected by QP optimization modeling.

Fresh pork and beef tend to be grouped together in the “red meat” or “non-poultry meat” category; yet they are very different in terms of cost, environmental impact, and nutritional value. The present analyses showed that once fresh pork was separated from beef, fresh pork was preferentially selected in a healthy low-cost diet for the Hispanic population. The amount of pork was higher in the H-TFP market basket as compared to the FP 2021. By contrast, an attempt to create vegetarian H-TFP failed to find a mathematical solution for several population subgroups. For the most part, the modeled food patterns did not supply adequate energy or were deficient in one or more nutrients.

There are some aspects of the TFP optimization modeling that need a more detailed discussion. Firstly, the TFP 2021 was based on a quadratic programming (QP) model [2]. QP modeling is used to generate food plans that meet energy, nutritional, and practicality requirements, subject to a number of social and cost constraints [5,6]. For the TFP 2021, optimization was constrained by the need to conform to the USDA Healthy US-Style Food Pattern, an example of an already optimized diet. Effectively, the CNPP QP model attempted to optimize an already optimized diet. For example, the minimum and maximum amounts of seafood and nuts and seeds were fixed by the USDA Healthy US-Style patterns and could not be changed.

Secondly, optimization models typically begin with the observed diet at the population level but that was not the starting point for the TFP 2021 optimization program; rather, the “observed” diets were also the healthiest diets, which are those with above-median HEI scores for each population subgroup. Diets with high HEI scores are necessarily biased in the direction of more vegetables, more whole grains, more seafood, and less solid fats, added sugars, and meat. While undoubtedly healthier, those are diets characteristic of only one half of the population. Starting with the population diet would most likely produce different TFP 2021 results. It would be good to take a hard look at some of the underlying assumptions that went into the creation of the TFP [25,26].

The optimization takes into account the energy and nutrient needs by gender and age group and estimates food groups and amounts based on dietary guidelines—that is the US-Style Healthy Food Plan. The objective function minimizes deviation from the existing eating patterns. Deciding on the “observed” diet has consequences for optimization modeling. For example, the existing US diet is very different from the demands of the Healthy US-Style Food Patterns and its requirements for seafood and close to five servings of fruit and vegetables per day. As a result, the deviation between the “observed” diet at the population level and the optimized will be quite high. Selecting diets that are closer to ideal as the “observed” starting point will reduce the deviation, but those diets are not necessarily representative of the population. Tailoring the optimized models to population subgroups will reduce the deviation.

The TFP also had some practicality constraints. Some items were fixed while others were not. For example, the amounts of coffee and tea were fixed. Given that a majority of adults in the United States consume coffee or tea, the market baskets for adults ages >20 y included a minimum of one cup per day of coffee or tea [2]. Otherwise, coffee and tea would not have been included in the TFP market basket.

By this time, several countries have developed methods to calculate the lowest cost of a healthy diet using a variety of methods. For example, the Australia Healthy Food Basket (HFB) method uses dietary guidelines and current food prices to calculate the cost of a healthy diet [27]. Canada’s National Nutritious Food Basket (NNFB) monitors the cost of a nutritious diet over time [28], whereas the UK Minimum Income Standard (MIS) includes food costs and the estimated cost of a healthy diet [29]. The Basic Food Basket in Brazil lists the minimum set of foods needed to meet nutrient requirements [30]. There has also been some US-based work on a more medically oriented TFP, tailored to the dietary needs of people with lactose intolerance, diets for persons with type 2 diabetes, and healthy budget-conscious diets that met pregnancy needs [31]. Based on some preliminary analyses, medically oriented market baskets in the US were all above the TFP cost (in 2019).

The present study had some features that can be perceived as weaknesses. Firstly, we followed the USDA methodology precisely, using diets with above-median HEI scores as the observed diets for the Hispanic population. Then, we calculated mean portion sizes in the manner outlined in the Supplemental files. We also kept the minimum and maximum food group constraints from the USDA Healthy US-Style food Pattern. We did not attempt to search for the lowest-cost food pattern; rather, the H-TFP was specifically intended to be cost-neutral. Acculturation of the Hispanic population was not addressed. There is some literature showing that eating habits change depending on place of birth and length of residence in the US. Attempts to model a cost-neutral vegetarian market basket for the TFP were not successful with the present set of constraints. One weakness of the current USDA approach to TFP modeling is that the observed diets were actually healthier than the norm and were not necessarily fully representative of the population.

On the other hand, the present analyses show how healthy and budget-conscious food plans can be developed for segments of the US population, including (potentially) populations with special medical or social needs. Optimized foods plans ought to take into account traditional diets that may include a higher proportion of fresh fruits, vegetables, and culturally specific ingredients. A more culturally sensitive approach to food assistance and nutrition programs is needed that would better address the diverse needs of different communities.

## 5. Conclusions

The modeled H-TFP illustrates how healthy food plans on a budget can be developed for specific subpopulations in the US. A growing segment of the US population that self-identifies as Hispanic appears to follow distinct food patterns that may reflect collective identity and culture. The optimized cost-neutral H-TFP market basket fixed at $186/week for a family of four selected fresh pork and reduced the amount of poultry. An alternative H-TFP market basket with pork as the only source of meat met nutrient requirements and practicality and cost constraints. By contrast, attempts to create a cost-neutral vegetarian H-TFP for the Hispanic population were unsuccessful and yielded no mathematical solution. Similar methods may also be used to develop healthy food plans on a budget for populations with special medical needs.

## Figures and Tables

**Table 1:** Summaries of 3 QP optimization models.

Protein Foods	Model 1 H-TFP	Model 2 H-TFP Pork	Model 3 Vegetarian
Modeling Categories			
Beef/pork	Separate	Separate	Separate
Beef	Defined by QP	Set to 0	Set to 0
Pork	Defined by QP	Set to meat + poultry in Model 1	Set to 0
Cured meat	Defined by QP	Set to 0	Set to 0
Poultry	Defined by QP	Set to 0	Set to 0
Seafood	Defined by QP	Defined by QP	Set to 0
Mixed dishes—meat, poultry, or seafood	Defined by QP	Defined by QP	Set to 0
Eggs, nuts, and soy	Defined by QP	Defined by QP	Defined by QP
Constraints			
Total cost	Same as USDA TFP	Same as USDA TFP	Same as USDA TFP
Min/max amounts from US-Style Food Pattern	Applied	Applied except for poultry and meat	Applied except for poultry, meat, seafood, meat/poultry/eggs, and total protein
Nutrients	Min/max amounts from DRIS for each age–gender group		
Objective function	Deviation from observed intakes weighted by cost—minimized		

**Table 2:** Characteristics of the Hispanic population in NHANES 2013–16. All percentages were survey weighted.

Variable	Modality	*n*	% Hispanic (N = 4180)	% Non-Hispanic (N = 9646)	*p*-Values (Chi2)
Hispanic origin	Mexican American	2586	63.0	0	
	Other Hispanic	1594	37.0	0	
Gender	Male	6750	49.3	49.6	<0.001
	Female	7076	50.67	50.4	
Reference person education level	Less than high school	2984	37.0	9.89	<0.001
	High school graduate	2950	22.0	19.7	
	Some college	4091	24.9	32.1	
	College graduate	3343	12.0	34.8	
	Refused/DK/missing	458	4.18	3.48	
Family income to poverty ratio (FIPR)	<1	3352	29.8	13.7	<0.001
	1–1.99	3343	27.2	18.1	
	2–3.49	2673	18.6	21.5	
	≥3.5	3399	13.9	41.2	
	Missing	1059	10.5	5.49	
Annual household income	$ 0 to $ 9999	826	6.91	3.71	<0.001
	$10,000 to $ 19,999	1540	12.2	6.97	
	$20,000 to $ 34,999	2529	20.6	12.9	
	$35,000 to $ 54,999	2430	20.0	16.1	
	$55,000 to $ 74,999	1463	10.5	11.8	
	$75,000 to $99,999	1309	9.02	11.5	
	$100,000 and over	2517	8.69	30.6	
	Refused/DK/missing	660	7.18	3.12	
Age–gender/group	Male 4–13 y	1703	10.2	7.18	<0.001
	Male 14–19 y	916	6.05	4.53	
	Male 20–50 y	2525	25.2	23.0	
	Male 51–70 y	1606	7.84	14.9	
	Female 4–13 y	1628	10.0	6.29	
	Female 14–19 y	938	6.22	4.41	
	Female 20–50 y	2802	26.3	23.3	
	Female 51–70 y	1708	8.16	16.4	

**Table 3:** Median and mean HEI-2015 vales for Hispanic and non-Hispanic populations.

Age–Gender/Group	Median HEI		Mean HEI		*p*-Value (*t*-Test)
	Hispanic	Non-Hispanic	Hispanic	Non-Hispanic	
Total sample	49.3	48.0	49.5	50.1	0.226
Male 4–13 y	48.7	46.3	49.0	47.3	0.072
Male 14–19 y	44.1	44.5	45.2	44.9	0.814
Male 20–50 y	47.0	47.7	47.8	48.7	0.368
Male 51–70 y	52.5	50.7	52.0	52.0	0.959
Female 4–13 y	48.9	46.7	49.6	47.6	0.044
Female 14–19 y	46.8	44.3	47.4	46.3	0.300
Female 20–50 y	50.3	48.9	50.5	51.0	0.454
Female 51–70 y	53.5	52.3	54.3	53.8	0.514

**Table 4:** Mean consumption (g/week) and mean costs ($/week) for a family of four by USDA combined food category. The data are based on the entire populations, not selected by HEI scores.

	Amount (g/Week)			Cost ($/Week)	
Combined Categories	Hispanic	Non-Hispanic	*p*-Value ^1^	Hispanic	Non-Hispanic
Total vegetables	2393	2442		12.42	13.95
Dark green vegetables	189	315	**	1.37	2.44
Red and orange vegetables	341	414	*	1.79	1.88
Beans, peas, lentils	466	93.8	****	1.43	0.29
Fried potato products	513	567	*	3.88	4.82
Starchy vegetables	205	294	ns	0.68	1.12
Other vegetables	679	760	ns	3.27	3.40
Total fruits	5153	4161		15.93	13.75
Whole fruit	2698	2396	ns	11.51	10.57
100% fruit juice	2455	1765	****	4.42	3.18
Total grains	3447	3606		15.95	19.1
Staple grains	2025	1854	*	7.15	7.76
Cereals	678	718	ns	2.76	2.76
Biscuits, muffins, quick breads	345	471	*	2.32	3.31
Popcorn	58.6	75.0	**	0.45	0.63
Tortilla, corn, other chips	210	228	ns	2.17	2.46
Crackers	89.6	164	**	0.73	1.42
Pretzels/snack mix	41.9	96.1	**	0.37	0.76
Total Dairy	5886	6289		10.8	13.37
Milk, yogurt, soy alternatives	5571	5853	ns	7.39	8.80
Cheese	316	437	***	3.41	4.57
Total protein foods	3368	3508		37.36	40.9
Beef	485	433	ns	11.05	9.51
Pork	139	154	*	1.68	1.87
Cured meat	399	665	****	4.86	8.82
Poultry	1107	1087	ns	10.24	10.28
Eggs	373	354	ns	1.32	1.28
Egg-based mixed dishes	360	225	*	1.68	0.97
Seafood	337	348	ns	4.42	5.34
Nut and seed	88.6	147	**	1.14	2.06
Nut and seed butters	29.4	59.5	***	0.16	0.34
Soy products	49.3	37.5	ns	0.81	0.43
Miscellaneous	29,685	30,579		97.66	95.09
Mixed dishes—beans, pulses	205.5	110.7	**	0.59	0.31
Mixed dishes—grain-based	4471	3268	****	24.23	16.44
Mixed dishes—meat, poultry, seafood	1031	1310	*	7.82	9.64
Pizza	1003	1167	*	8.24	10.01
Sandwiches	1384	1256	*	8.96	9.11
Mixed dishes—vegetables	550	838	**	2.56	3.89
Soups	1508	1046	*	4.85	2.85
Coffee and tea	5298	6957	**	4.95	4.62
Butter and animal fats	29.4	31.0	***	0.29	0.28
Margarine, oils, cream	302	397	**	1.49	2.05
Condiments and sauces	460	482	*	2.32	2.74
Sugar and sugar substitutes	168	203	*	0.75	0.85
Sodas	8364	8387	*	10.17	10.32
Fruit drinks	2242	2268	ns	2.51	2.40
Other beverages	704	630	ns	2.20	2.37
Sweet bakery products	1021	987	ns	9.31	8.22
Snack bars	73.8	106	*	1.02	1.56
Candy	206	352	****	2.43	4.03
Other desserts	664	788	ns	2.97	3.40
TOTAL	49,932	50,585		190.12	196.16

^1^ *t*-Test have been performed on each of the four age–gender group (male aged 20–50 years, female aged 20–50 years, male aged 4–13 years, and female aged 4–13 years) in the family of four. The number of asterisks (from 0 to 4) indicates the number of age–gender groups. ns: not significant for all four age–gender groups.

**Table 5:** Mean estimated portion sizes (g/week) and mean costs ($/week) of a family of four for diets above the median HEI-2015 values for the Hispanic and non-Hispanic populations by USDA food group and food category.

	Amount (g/Week)			Cost ($/Week)	
Combined Categories	Hispanic	Non-Hispanic	*p*-Value ^1^	Hispanic	Non-Hispanic
Total vegetables	2848	2808		14.25	15.8
Dark green vegetables	258.0	456.8	**	1.87	3.54
Red and orange vegetables	389.2	536.0	**	2.04	2.44
Beans, peas, lentils	753.9	168.6	****	2.31	0.51
Fried potato products	245.1	346.7	ns	0.82	1.32
Starchy vegetables	523.4	542.7	ns	3.95	4.61
Other vegetables	678.1	757.1	ns	3.26	3.38
Total fruits	4861	3638		12.86	10.55
Whole fruit	1667	1531	ns	7.11	6.75
100% fruit juice	3194	2108	***	5.75	3.80
Total grains	2120	2221		10.54	12.61
Staple grains	1049	923.5	*	3.70	3.86
Cereals	446.4	465.1	ns	1.81	1.79
Biscuits, muffins, quick breads	330.8	393.1	ns	2.22	2.77
Popcorn	33.5	39.5	*	0.26	0.33
Tortilla, corn, other chips	185.0	222.6	ns	1.92	2.41
Crackers	41.9	80.1	*	0.34	0.69
Pretzels/snack mix	33.4	96.8	***	0.29	0.76
Total dairy	2815	3155		6.34	8.07
Milk, yogurt, soy alternatives	2541	2784	ns	3.37	4.19
Cheese	274.9	370.9	ns	2.97	3.88
Total protein foods	2502	2687		26.83	30.78
Beef	225.4	204.8	ns	5.13	4.50
Pork	86.1	73.4	ns	1.04	0.89
Cured meat	311.2	547.7	**	3.80	7.27
Poultry	594.4	580.3	ns	5.50	5.49
Eggs	401.0	379.0	ns	1.42	1.37
Egg-based mixed dishes	178.2	112.3	ns	0.83	0.49
Seafood	516.3	506.1	ns	6.76	7.77
Nut and seed	75.1	127.1	**	0.97	1.78
Nut and seed butters	45.3	98.5	**	0.24	0.56
Soy products	69.0	57.4	ns	1.14	0.66
Miscellaneous	13,586	14,244		50.26	50.74
Mixed dishes—beans, pulses	133.5	86.1	*	0.38	0.24
Mixed dishes—grain-based	1765	1496	*	9.57	7.53
Mixed dishes—meat, poultry, seafood	579.6	725.6	ns	4.40	5.34
Pizza	307.8	335.1	ns	2.53	2.88
Sandwiches	492.0	473.7	ns	3.18	3.44
Mixed dishes—vegetables	297.1	434.1	*	1.38	2.02
Soups	637.2	482.2	*	2.05	1.31
Coffee and tea	2172	2916	**	2.03	1.94
Butter and animal fats	29.4	29.4	***	0.29	0.26
Margarine, oils, cream	250.7	364.3	**	1.24	1.88
Condiments and sauces	365.3	375.9	*	1.84	2.14
Sugar and sugar substitutes	150.3	165.1	ns	0.67	0.69
Sodas	2867	2879	*	3.49	3.54
Fruit drinks	910.3	803.0	*	1.02	0.85
Other beverages	1033	1034	ns	3.22	3.88
Sweet bakery products	787.4	640.1	*	7.18	5.33
Snack bars	102.3	134.9	*	1.41	1.99
Candy	166.6	242.4	*	1.97	2.78
Other desserts	538.4	626.3	*	2.41	2.70
TOTAL	28,732	28,753		121.08	128.55

^1^ *t*-Test have been performed on each of the four age–gender group (male aged 20–50 years, female aged 20–50 years, male aged 4–13 years, and female aged 4–13 years) in the family of four. The number of asterisks (from 0 to 4) indicates the number of age–gender groups for which the *p*-value of the *t*-test was significant. ns: not significant for all age–gender groups.

**Table 6:** Modeled food patterns for the USA population1 and H-TFP Model 1 and Model 2 (pork only). The optimization follows the USDA approach—starting with the observed serving sizes. Amounts in g/week and costs in $/week are for a family of four.

	TFP 2021 (US Old) ^1^	Model 1 H-TFP	Model 2 H-TFP Pork	TFP 2021 (US Old) ^1^	Model 1 H-TFP	Model 2 H-TFP Pork
Market Basket	Amount, g/Week	Amount, g/Week	Amount, g/Week	Cost, $/Week	Cost, $/Week	Cost, $/Week
Total vegetables	10,962	11,020	11,013	40.19	40.35	41.08
Dark green vegetables	1155	1198	1195	5.67	5.92	5.91
Red and orange vegetables	2795	2800	2848	9.64	10.05	10.13
Beans, peas, lentils	1720	1686	1663	5.26	5.15	5.04
Starchy vegetables	2608	2870	2782	10.65	11.16	10.91
Other vegetables	2684	2466	2525	8.97	8.07	9.09
Total fruits	10,734	10,445	10,828	27.73	26.08	27.05
Whole fruit	5356	4645	4824	17.94	15.99	16.61
100% fruit juice	5378	5800	6004	9.79	10.09	10.44
Total grains	8627	10,461	9675	28.2	29.42	30.46
Whole-grain rice, pasta, breads, tortillas	3441	2665	2943	14.76	11.07	12.26
Whole-grain cereals (oatmeal, RTE cereal)	2350	5374	4035	5.58	10.42	7.83
Refined-grain rice, pasta, breads, tortillas	2698	2138	1987	6.78	5.28	4.83
Refined-grain cereals, crackers, snacks	138	284	710	1.08	2.65	5.54
Total dairy	16,381	11,405	14,818	29.12	27.05	25.49
Lower nutrient density milk, yogurt, soy	4840	0	1500	7.24	0	2.05
Higher nutrient density milk, yogurt, soy	10,551	9766	12,336	13.74	10.88	13.75
Cheese	990	1639	982	8.14	16.17	9.69
Total protein foods	6499	6406	6246	46.69	46.64	49.76
Beef	0	0	0	0	0	0.00
Pork	928	1285	3259	6.9	11.27	28.54
Cured meat	0	0	0	0	0	0.00
Poultry	2573	1974	0	18.26	13.57	0.00
Eggs	904	1034	872	3.29	3.69	3.12
Seafood	1235	1223	1223	13.38	13.21	13.21
Nuts, seeds, soy products	859	890	892	4.86	4.9	4.89
Miscellaneous	7846	10,032	7999	17.99	20.35	16.07
RTE soups, entrees, pizza, side dishes	3861	3866	3743	12.73	11.36	11.70
Coffee and tea	3360	3360	3360	1.75	1.8	1.80
Table fats and oils	346	185	351	1.86	1.02	1.83
Sauces, condiments, sweets, spices	61	182	15	0.26	0.8	0.07
Other misc. foods and beverages	218	2439	530	1.39	5.37	0.67
Total	61,049	59,769	60,579	189.91	189.91	189.91

^1^ https://www.mdpi.com/2072-6643/15/8/1897 (accessed on 24 July 2024).

## Data Availability

Publicly available datasets were analyzed in this study. These data can be found at: https://www.fns.usda.gov/cnpp/thrifty-food-plan-2021 (accessed on 24 July 2024), https://www.ars.usda.gov/northeast-area/beltsville-md-bhnrc/beltsville-human-nutrition-research-center/food-surveys-research-group/docs/wweianhanes-overview/ (accessed on 24 July 2024), https://wwwn.cdc.gov/nchs/nhanes/continuousnhanes/overview.aspx?BeginYear=2015 (accessed on 24 July 2024), https://www.ars.usda.gov/northeast-area/beltsville-md-bhnrc/beltsville-human-nutrition-research-center/food-surveys-research-group/docs/fped-overview/ (accessed on 24 July 2024), https://epi.grants.cancer.gov/hei/ (accessed on 24 July 2024). The original contributions presented in the study are included in the article/Supplementary Material; further inquiries can be directed to the corresponding author.

## Supplementary Materials

The following supporting information can be downloaded at: https://www.mdpi.com/article/10.3390/nu16172915/s1, Table S1: categorization of market basket, combined, TFP, and modeling categories; Table S2: prices ($/100 g), nutrient density scores (%/100 kcal), and energy density (kcal/100 g) for high-cost and low-cost items for protein foods for the Hispanic population. Nutrient density scores based on standard for male, aged 20–50 y; Table S3: total costs in dollars per week and dietary deviations for age gender groups by model type.
