# Supplementary material for: Hispanic Thrifty Food Plan (H-TFP): Healthy, Affordable, and Culturally Relevant
Source: Nutrients. 2024 Sep 1;16(17):2915. doi: 10.3390/nu16172915 (PMC11397355; doi:10.3390/nu16172915)
Supplement: Supplementary file 1 [file nutrients-16-02915-s001.zip › TABLE_S1_Nutrients_Submitted.pdf]

Supplemental Table S1: Categorization of Market Basket, combined, TFP and modeling categories

**Market basket category**	**Combined category (n=46)**	**Initial category[1] (n=67)**	**Modeling category (n=99)**
Vegetables			
Dark green vegetables	Dark green vegetables	Dark green vegetables	Dark green vegetables, HC[2]
			Dark green vegetables, LC[3]
Red and orange vegetables	Red & orange vegetables	Red & orange vegetables	Red & orange vegetables, HC
			Red & orange vegetables, LC
Beans, peas, lentils	Beans, peas, lentils	Beans, peas, lentils	Beans, peas, lentils
Starchy vegetables	Starchy vegetables	Starchy vegetables	Starchy vegetables, HC
			Starchy vegetables, LC
	Fried Potato Products	Fried Potato Products	Fried Potato Products
Other vegetables	Other vegetables	Other vegetables	Other vegetables, HC
			Other vegetables, LC
Fruits			
Whole fruit	Fruit	Fruit, HND[4] (0 g added sugars/100 g and/or <0.5 g saturated fat/100 g)	Fruit, HND, HC
			Fruit, HND, LC
		Fruit, LND[5]	Fruit, LND, HC
			Fruit, LND, LC
100% fruit juice	100% juice	100% juice	100% juice, HC
			100% juice, LC
Grains			
Whole grains, staple grains	Staple grains	Staple grains, HND (≥50% whole grains)	Staple grains, HND, HC
			Staple grains, HND, LC
Refined grains , staples grains		Staple grains, LZD	Staple grains, LND, HC
			Staple grains, LND, LC
	Biscuits, muffins, quick breads	Biscuits, muffins, quick breads	Biscuits, muffins, quick breads
Whole grains, staple grains	Popcorn	Popcorn, HND (0 g added sugars/100 g and <7 g saturated fat/100 g)	Popcorn, HND
		Popcorn, LND	Popcorn, LND

Whole grains, cereals	Cereals	Cereals, HND (≤21.2 g total sugars/100 g and ≥50% whole grains)	Cereals, HND
Refined grains, cereals		Cereals, LND	Cereals, LND
Refined grain other	Tortilla, corn, other chips	Tortilla, corn, other chips	Tortilla, corn, other chips
	Crackers	Crackers, HND (≥50% whole grains)	Crackers, HND
		Crackers, LND	Crackers, LND
	Pretzels/snack mix	Pretzels/snack mix	Pretzels/snack mix
Dairy			
Higher nutrient density milk, yogurt, soy alternatives	Milk and Yogurt	Milk and Yogurt, HND (plain/flavored nonfat/1% milk, milk and non-whole milk yogurt with 0 g added sugars/100 g, soy beverage with <3.3 g added sugars/100 g)	Milk and Yogurt, HND
Lower nutrient density milk, yogurt, soy alternatives		Milk and Yogurt, LND	Milk and Yogurt, LND
Cheese	Cheese	Cheese	Cheese, HC
			Cheese, LC
Protein foods			
Pork	Pork	Pork, HND (≤4.5 g saturated fat/100 g and 0 g added sugars/100 g)	Pork, HND, HC
			Pork, HND, LC
		Pork, LND	Pork, LND, HC
			Pork, LND, LC
Beef	Beef	Beef, HND (≤4.5 g saturated fat/100 g and 0 g added sugars/100 g)	Beef, HND, HC
			Beef, HND, LC
		Beef, LND	Beef, LND, HC
			Beef, LND, LC
Cured meat	Cured meat	Cured meat	Cured meat
Poultry	Poultry	Poultry, HND (≤4.5 g saturated fat/100 g and 0 g added sugars/100 g)	Poultry, HND, HC
			Poultry, HND, LC
		Poultry, LND	Poultry, LND, HC
			Poultry, LND, LC

Eggs	Eggs	Eggs	Eggs
	Mixed Dishes - Eggs	Mixed Dishes – Eggs, HND (< median sodium content/100 g in WWEIA category)	Mixed Dishes – Eggs, HND
		Mixed Dishes – Eggs, LND	Mixed Dishes – Eggs, LND
Seafood	Seafood	Seafood	Seafood, HC
			Seafood, LC
Nuts, seeds, soy products	Nut & seed butters	Nut & seed butters	Nut & seed butters
	Nuts & Seeds	Nuts & Seeds, HND (0 g added sugars/100 g)	Nuts & Seeds, HND
		Nuts & Seeds, LND	Nuts & Seeds, LND
	Processed soy products	Processed soy products	Processed soy products
Miscellaneous			
Ready-to-eat and ready-to-heat	Mixed Dishes - Beans, peas, lentils	Mixed Dishes- Beans, peas, lentils, HND (< median sodium content/100 g in WWEIA category)	Mixed Dishes - Beans, peas, lentils, HND, HC
			Mixed Dishes - Beans, peas, lentils, HND, LC
		Mixed Dishes - Beans, peas, lentils, LND	Mixed Dishes - Beans, peas, lentils, LND, HC
			Mixed Dishes - Beans, peas, lentils, LND, LC
	Mixed Dishes - Grain based	Mixed Dishes- Grain based, HND (< median sodium content/100 g in WWEIA category)	Mixed Dishes - Grain based, HND, HC
			Mixed Dishes - Grain based, HND, LC
		Mixed Dishes - Grain based, LND	Mixed Dishes - Grain based, LND, HC
			Mixed Dishes - Grain based, LND, LC
	Mixed Dishes - Meat, Poultry, Seafood	Mixed Dishes- Meat, Poultry, Seafood, HND (< median sodium content/100 g in WWEIA category)	Mixed Dishes - Meat, Poultry, Seafood, HND, HC
			Mixed Dishes - Meat, Poultry, Seafood, HND, LC
		Mixed Dishes - Meat, Poultry, Seafood, LND	Mixed Dishes - Meat, Poultry, Seafood, LND, HC
			Mixed Dishes - Meat, Poultry, Seafood, LND, LC
	Pizza	Pizza, HND	Pizza, HND, HC
			Pizza, HND, LC

		(< median sodium content/100 g in WWEIA category)	
		Pizza, LND	Pizza, LND, HC
			Pizza, LND, LC
	Mixed Dishes - Sandwiches	Mixed Dishes- Sandwiches, HND (< median sodium content/100 g in WWEIA category)	Mixed Dishes - Sandwiches, HND, HC
			Mixed Dishes - Sandwiches, HND, LC
		Mixed Dishes - Sandwiches, LND	Mixed Dishes - Sandwiches, LND, HC
			Mixed Dishes - Sandwiches, LND, LC
	Mixed Dishes - Vegetables	Mixed Dishes- Vegetables, HND (< median sodium content/100 g in WWEIA category)	Mixed Dishes - Vegetables, HND, HC
			Mixed Dishes - Vegetables, HND, LC
		Mixed Dishes - Vegetables, LND	Mixed Dishes - Vegetables, LND, HC
			Mixed Dishes - Vegetables, LND, LC
	Soups	Soups, HND (< median sodium content/100 g in WWEIA category)	Soups, HND, HC
			Soups, HND, LC
		Soups, LND	Soups, LND, HC
			Soups, LND, LC
Coffee & Tea	Coffee & Tea	Coffee & Tea, HND (0 g added sugars/100 g)	Coffee & Tea, HND
		Coffee & Tea, LND	Coffee & Tea, LND
Table fat and oils	Butter and animal fats	Butter and animal fats	Butter and animal fats
	Margarine, oils, cream, cream substitutes	Margarine, oils, cream, cream substitutes	Margarine, oils, cream, cream substitutes
Sauces, condiments, jams, honey, sugars, spices	Condiments and Sauces	Condiments and Sauces	Condiments and Sauces, HC
			Condiments and Sauces, LC
	Sugar and sugar substitutes	Sugar and sugar substitutes	Sugar and sugar substitutes
Other Miscellaneou	Sodas	Diet beverages	Diet beverages
		Soft drinks	Soft drinks

s Foods and Beverages	Fruit drinks	Fruit drinks, HND (0 g added sugars/100 g)	Fruit drinks, HND
		Fruit drinks, LND	Fruit drinks, LND
	Milk substitutes, nutritional beverages, and smoothies	Milk substitutes, nutritional beverages, and smoothies	Milk substitutes, nutritional beverages, and smoothies
	Sweet bakery products	Sweet bakery products	Sweet bakery products
	Snack Bars	Snack Bars	Snack Bars
	Candy	Candy	Candy
	Other Desserts	Other Desserts	Other Desserts

[1]Inclusion criteria for higher nutrient density category detailed in brackets.[2]HC: Hight Cost. [3]LC : Low cost. [4]HND : Hight nutrient density. [5]LND : Low nutrient density.
