# Supplementary material for: Hispanic Thrifty Food Plan (H-TFP): Healthy, Affordable, and Culturally Relevant
Source: Nutrients. 2024 Sep 1;16(17):2915. doi: 10.3390/nu16172915 (PMC11397355; doi:10.3390/nu16172915)
Supplement: Supplementary file 1 [file nutrients-16-02915-s001.zip › TABLE_S2_Nutrients_Submitted.pdf]

Supplemental Table S2. Prices ($/100g), nutrient density scores (%/100kcal), and energy density (kcal/100g) for high cost and low cost items for protein foods for the Hispanic population. Nutrient density scores based on standard for male, aged 20-50y.

Protein foods	Modeling category	n	Price ($/100 g)	NDS24 (%/100 kcal)	Energy (kcal/100 g)
Dairy	High cost	1	1.23	5.12	341
	Low cost	1	0.99	5.75	314
	Other	2	0.12	8.68	49.6
Protein foods					
Beef	High cost	2	2.41	**8.05**	219
	Low cost	2	1.36	**6.86**	235
Pork	High cost	2	1.43	**5.82**	291
	Low cost	2	0.96	**7.54**	222
Poultry	High cost	2	1.22	5.53	219
	Low cost	2	0.73	5.69	202
Seafood	High cost	1	1.72	**9.82**	164
	Low cost	1	1.08	**8.25**	150
Cured meat	Other	1	1.22	5.73	200
Eggs	Other	3	0.43	6.78	187
Nuts, seeds, soy	Other	4	1.13	6.20	485
