# Supplementary material for: Hispanic Thrifty Food Plan (H-TFP): Healthy, Affordable, and Culturally Relevant
Source: Nutrients. 2024 Sep 1;16(17):2915. doi: 10.3390/nu16172915 (PMC11397355; doi:10.3390/nu16172915)
Supplement: Supplementary file 1 [file nutrients-16-02915-s001.zip › TABLE_S3_Nutrients_Submitted.pdf]

Supplemental Table S3. Total costs in dollars per week and dietary deviations for age gender groups by model type.

	Costs per week			Dietary deviation in g/day		
**Age-sex group**	**TFP and H-TFP cost $**	**H-TFP Vegetarian**		**H-TFP**	**Model 2 H-TFP pork**	**H-TFP Vegetarian**
Family of 4	189.91	infeasible		273	346	infeasible
Male 4-13y	42.07	42.07		58	77	82
Male 14-19y	61.46	infeasible		90	102	infeasible
Male 20-50y	59.78	infeasible		86	89	infeasible
Male 51-70y	52.36	infeasible		80	84	infeasible
Female 4-13y	40.25	40.25		64	90	94
Female 14-19y	48.72	infeasible		94	101	infeasible
Female 20-50y	47.81	47.81		64	96	113
Female 51-70y	44.38	44.38		91	76	113
